# Pronounced α-Synuclein Pathology in a Seeding-Based Mouse Model Is Not Sufficient to Induce Mitochondrial Respiration Deficits in the Striatum and Amygdala

**DOI:** 10.1523/ENEURO.0110-20.2020

**Published:** 2020-08-14

**Authors:** Johannes Burtscher, Jean-Christophe Copin, Carmen Sandi, Hilal A. Lashuel

**Affiliations:** 1 Laboratory of Molecular and Chemical Biology of Neurodegeneration, Brain Mind Institute, École Polytechnique Fédérale de Lausanne, Lausanne, CH-1015, Switzerland; 2 Laboratory of Behavioral Genetics, Brain Mind Institute, EPFL, Lausanne, CH-1015, Switzerland

**Keywords:** amygdala, mitochondria, neurodegeneration, Parkinson’s disease, synuclein

## Abstract

Increasing evidence suggests that cross talk between α-synuclein pathology formation and mitochondrial dysfunction plays a central role in the pathogenesis of Parkinson’s disease (PD) and related synucleinopathies. While mitochondrial dysfunction is a well-studied phenomenon in the substantia nigra, which is selectively vulnerable in PD and some models thereof, less information is available in other brain regions that are also affected by synuclein pathology. Therefore, we sought to test the hypothesis that early α-synuclein pathology causes mitochondrial dysfunction and that this effect might be exacerbated in conditions of increased vulnerability in affected brain regions, such as the amygdala. We combined a model of intracerebral α-synuclein pathology seeding with chronic glucocorticoid treatment, which models non-motor symptoms of PD and affects amygdala physiology. We measured mitochondrial respiration, reactive oxygen species (ROS) generation and protein abundance as well as α-synuclein pathology in male mice. Chronic corticosterone administration induced mitochondrial hyperactivity in the amygdala. Although injection of α-synuclein preformed fibrils (PFFs) into the striatum resulted in pronounced α-synuclein pathology in both striatum and amygdala, mitochondrial respiration in these brain regions was not compromised, regardless of corticosterone treatment. Our results suggest that early stage α-synuclein pathology does not influence mitochondrial respiration in the striatum and amygdala, even in corticosterone-induced respirational hyperactivity. We discuss our findings in light of relevant literature, warn of a potential publication bias and encourage scientists to report their negative results within the framework of this model.

## Significance Statement

We report that early stage α-synuclein pathology by itself or in combination with exogenous corticosterone-induced amygdala hyperactivity did not compromise mitochondrial respiration in the striatum and amygdala in one of the most commonly used models of synucleinopathies. These results may explain why, in the hands of many research groups, this model does not elicit pronounced Parkinson’s disease (PD)-like symptoms in the absence of mitochondrial dysfunction. This despite the presence of significant α-synuclein pathology in brain regions involved in non-motor (amygdala) and motor (striatum) disease symptoms. Our findings call for rigorous investigation of the short- and long-term effects of α-synuclein pathology on mitochondrial function/dysfunction in this model, particularly in brain regions strongly affected by neurodegeneration such as the substantia nigra pars compacta.

## Introduction

The misfolding, aggregation and accumulation of α-synuclein in Lewy bodies and the selective neurodegeneration of dopaminergic neurons are defining hallmarks of Parkinson’s disease (PD), the most common neurodegenerative motor disease (Spillantini et al., 1997; Lashuel et al., 2013). Mutations or multiplications of the α-synuclein encoding gene, *SNCA*, cause familial forms of PD (Polymeropoulos et al., 1997; Krüger et al., 1998; Singleton et al., 2003; Ibáñez et al., 2004; Zarranz et al., 2004), thereby suggesting a causal role of α-synuclein in PD. Several lines of evidence support the Braak hypothesis of α-synuclein pathology propagation during the progression of PD (Braak et al., 2003a,b), including evidence for (1) pathologic α-synuclein spreading from host PD patient brains to mesencephalic transplants grafted into these brains (Kordower et al., 2008; Li et al., 2008); (2) intercellular transmission of α-synuclein aggregates (Desplats et al., 2009; Volpicelli-Daley et al., 2011); and (3) induction of α-synuclein pathology formation and spreading in mouse brain by inoculation with recombinant α-synuclein preformed fibrils (PFFs) or brain-derived aggregates from PD or multiple system atrophy (MSA) patients (Luk et al., 2012; Masuda-Suzukake et al., 2013; Recasens et al., 2014).

Mitochondrial dysfunctions, in particular deficiencies of the electron transport system, have been implicated in PD pathogenesis (Langston et al., 1983; Schapira et al., 1989) and is thought to play important roles in both neurodegeneration and α-synuclein pathology formation (Hsu et al., 2000). Aggregated forms of α-synuclein have also been shown to interfere with the regulation of mitochondrial import (Di Maio et al., 2016), mitochondrial membrane potential, reactive oxygen species (ROS) generation and mitochondrial morphology (Tapias et al., 2017; Grassi et al., 2018) and oxidative phosphorylation (Devi et al., 2008; Ludtmann et al., 2016, 2018; Mahul-Mellier et al., 2020). However, whether α-synuclein pathology in the brain is sufficient to cause mitochondrial respiration dysfunction is not known. Animal models based on α-synuclein pathology seeding provide unique opportunities to address this question.

Seeding and induction of α-synuclein pathology in rodents by inoculation of the striatum (Luk et al., 2012) or the olfactory bulb (Rey et al., 2016) with PFFs results in robust early α-synuclein pathology (as assessed by staining for α-synuclein phosphorylated at serine 129, pS129) in the amygdala (Burtscher et al., 2019) preceding motor symptoms. We reported a peak of pS129 α-synuclein in limbic and cortical circuits around one to three months after injection that subsequently decreased over time. Such transiently high levels of pS129 α-synuclein especially in the amygdala (Burtscher et al., 2019), although potentially mouse-strain dependent, are increasingly acknowledged by some groups (Rey et al., 2019) but not yet by others (Henderson et al., 2019).

As the human amygdala can also be profoundly affected in synucleinopathy (Nelson et al., 2018; Sorrentino et al., 2019), we initially thought such pathology could be related to prodromal and non-motor symptoms in PD. However, we recently reported surprisingly small behavioral effects associated with high levels of pathologic α-synuclein in the mouse amygdala (Burtscher et al., 2019), suggesting that α-synuclein pathology in the amygdala was not sufficient to cause major non-motor symptoms. Whether this is due to the high resistance of cells in the amygdala to the cellular stress conferred by pathologic α-synuclein aggregates, or whether creeping cellular dysfunction at this stage precedes behavioral phenotypes is unknown. We hypothesized here that early α-synuclein pathology perturbs mitochondrial function and thus causes detectable cellular dysfunction that precedes behavioral manifestation.

To investigate this hypothesis, we injected α-synuclein PFFs into the striatum of adult male mice. Based on our previous results (Burtscher et al., 2019), we selected to study mitochondrial function at a time point where α-synuclein pathology peaks in the amygdala, which was five to six weeks after injection (Burtscher et al., 2019). We characterized pathology using biochemistry and imaging approaches at this time point and studied mitochondrial functions using high-resolution respirometry and concurrent assessment of mitochondrial ROS generation. All these assessments were performed for the amygdala and the site of injection, the striatum. To exclude the potential of the results being confounded by cellular loss-induced mitochondrial dysfunction, we did not investigate time points or brain regions characterized by apparent neuronal cell death.

In addition, we applied chronic corticosterone (in the drinking water) to a subset of mice to induce metabolic dysfunction in the amygdala, starting one month before PFF injection and until the animals were killed. Metabolic alterations in the amygdala have been reported on chronic glucocorticoid administration (Thobois et al., 2017). This approach allowed us to assess the influence of preexisting metabolic dysfunction in combination with α-synuclein pathology on mitochondrial function.

## Materials and Methods

### PFFs

α-Synuclein fibrils were prepared and characterized as described previously (Burtscher et al., 2019; Kumar et al., 2020). Briefly, a 325 μm solution of pure recombinant mouse α-synuclein protein (in PBS, pH 7.2) was centrifuged in 0.2 μm filter tubes (5 min, 5000 rpm). To produce fibrils (PFFs), the supernatant was incubated under aggregation conditions (4 d, 37°C) and shaking (at 900 rpm), as described previously (Burtscher et al., 2019). The resulting PFFs were sonicated (one 1-s pulse per 2 s for 10 s at 40% amplitude) and stored at −80°C until use.

For characterization of the PFFs, samples were treated with 10 μm thioflavin T (ThT; in 50 mm glycine, pH 8.5) in black 384-well plates (Nunc) and the extent of fibril formation was measured at 485 nm (excitation at 450 nm) using a Bucher Analyst AD plate reader.

To assess the amount of fibrils formed and the monomers released, we used sedimentation (100,000 × *g*, 30 min) and filtration assays (14,000 × *g* for 15 min through a 100-kDa filter). Electron microscopy was applied for morphologic characterizations, as described previously (Burtscher et al., 2019; Kumar et al., 2020).

### Animals

Male C57BL/6JRj mice (two to three months of age, three animals per cage) used for the in vivo experiments were housed at 23°C, 40% humidity, light from 7 A.M. to 7 P.M. and dark from 7 P.M. to 7 A.M. with free access to standard laboratory rodent chow and water for *in vivo* experiments. Primary hippocampal cultures were derived from postnatal day (P) P0-2 pups of C57BL/6JRccHsd mice.

All animal experimentation procedures were approved by the Cantonal Veterinary Authorities (Vaud, Switzerland) and were performed in compliance with the European Communities Council Directive of 24 November 1986 (86/609EEC). Every effort was taken to minimize the number of the animals and their stress.

For stereotactic surgeries, 5 μg of PFFs in 2 μl PBS was injected into the right dorsal striatum (AP +0.4, ML +2, DV −2.6) of fully anesthetized animals (100 mg/kg ketamine and 10 mg/kg xylazine, i.p.) on stereotactic frames (Kopf Instruments) through a 34-gauge cannula using a 10-μl Hamilton syringe (flow rate of 0.1 μl/min).

Mice were given an overdose of pentobarbital (150 mg/kg) for transcardial perfusion with heparinized saline, which was followed by 4% paraformaldehyde perfusion to prepare the tissue for histology (*N* = 3 per group, total *N* = 24). Mice for mitochondrial respiration studies (*N* = 9 per group, *N* = 36) were killed by neck dislocation, and exsanguination and tissues were directly dissected and prepared for high-resolution respirometry.

### Primary neuronal cultures, PFF treatment, and immunocytochemistry

Primary hippocampal neurons were prepared from P0-2 C57BL/6JRccHsd mouse pups (Harlan) and were imaged as described before (Mahul-Mellier et al., 2020). Briefly, hippocampi were dissected in HBSS and digested by papain (20 U/ml, Sigma-Aldrich) for 30 min at 37 °C. Papain activity was inhibited using a trypsin inhibitor (Sigma-Aldrich), and tissues were dissociated by trituration. The cells were resuspended in adhesion media (MEM, 10% horse serum, 30% glucose, L-glutamine, and penicillin/streptomycin; Life Technologies), and they were plated at a density of 250,000 cells/ml in six-well plates coated with 0.1% (w/v) poly-L-lysine in water (Brunschwig). Adhesion media were replaced by neurobasal medium (Life Technologies) containing B27 supplement (Life Technologies), L-glutamine, and penicillin/streptomycin (100 U/ml, Life Technologies) after 3 h. After 5 d in vitro, primary cultures were treated with 70 nm (corresponding to ∼1 ng/μl) PFFs; 14 d later, the cells were washed in PBS, fixed in 4% PFA for 20 min, and immunostained. For Mito Tracker experiments, the cells were exposed to 100 nm MitoTracker Red CMXRos (Invitrogen) 30 min before fixation. For immunostaining, fixed cells were blocked with 3%BSA with 0.1% Triton X-100 in PBS for 30 min, and then they were exposed to primary antibodies for 2 h, washed, and incubated with secondary antibodies (plus DAPI) for 1 h, washed and mounted using Fluoromount (Southern Biotech).

### Assessment of body composition

Percentages of fat and lean masses of total body mass were measured by echo MRI before corticosterone/vehicle treatment (to ensure similar body composition across experimental groups) and eight weeks after treatment.

### Continuous exogenous corticosterone treatment

Corticosterone (Sigma) was dissolved in 0.45% hydroxypropyl-b-cyclodextrin (Sigma) and administered (35 mg/l) to animals in drinking water continuously starting four weeks before surgery and continuing until the killing of the animals as described previously (Bacq et al., 2012). Control animals were administered the solvent vehicle (0.45% hydroxypropyl-b-cyclodextrin) in the same manner.

### Histology and immunohistochemistry

Perfused brains were postfixed in 4% paraformaldehyde, embedded in paraffin, and cut coronally to generate 4-μm sections. Brain slices were dewaxed and epitope retrieval was performed for 20 min at 95°C in trisodium citrate buffer (10 mm, pH 6.0) in a retriever (Labvision) was applied. For immunofluorescence, sections were blocked for 60 min in 3% bovine serum albumin in PBS containing 0.1% Triton X-100. Primary antibodies were applied overnight at 4°C, followed by secondary antibodies (plus DAPI) for 60 min at room temperature (RT) before mounting the slides using Fluoromount (Southern Biotech). Sections of *N* = 3 animals from each time point [30  d postinjection (dpi) and 360 dpi of only PFFs or PBS, total *N* = 12] were used for pS129 staining together with ubiquitin, p62 or thioflavin S (ThS). For ThS staining, sections were incubated for 15 min in 0.01% ThS and washed in 80% ethanol, followed by washing in water and then PBS before blocking and immunostaining.

Sections of *N* = 3 animals per group (total *N* = 12) at 60 dpi (corticosterone/vehicle and PFF/PBS) were used for staining for pS129, N-terminal (1–20) α-synuclein and TOM20, with or without proteinase K treatment (8 min in 1 μg/ml of proteinase K in 50 mm Tris-HCl buffer at pH 7.4 before staining). Imaging was performed with a Zeiss LSM700 confocal microscope.

Other sections of the same animals were used for 3,3’-diaminobenzidine (DAB)-staining and exposed to 3% H_2_O_2_ in PBS for 30 min before blocking for 60 min in 3% bovine serum albumin in PBS containing 0.1% Triton X-100 at RT. A primary antibody against α-synuclein pS129 (Wako Chemicals USA, 014-20281, 1:10,000) was incubated with the sections overnight at 4°C, and ImmPRESS reagent anti-mouse IgG was applied for 40 min at RT followed by incubation for 10 min in DAB dissolved in 50 mm Tris buffer and 0.06% H_2_O_2_. Sections were counterstained with Mayer’s hematoxylin, mounted with Fluoromount (Southern Biotech), and imaged using an Olympus AX70 microscope.

### Respirometry

After killing, striatum and amygdala-enriched tissues (both from the hemisphere of injection and contralateral hemisphere) were dissected on ice using a mouse brain matrix (Agnthos). Wet tissue was weighed and collected in ice-cold BIOPS [2.8 mm Ca_2_K_2_EGTA, 7.2 mm K_2_EGTA, 5.8 mm ATP, 6.6 mm MgCl_2_, 20 mm taurine, 15 mm sodium phosphocreatine, 20 mm imidazole, 0.5 mm dithiothreitol, and 50 mm MES; pH 7.1]. Then, the tissue was homogenized in ice-cold MiR05 [0.5 mm EGTA, 3 mm MgCl_2_, 60 mm potassium lactobionate, 20 mm taurine, 10 mm KH_2_PO_4_, 20 mm HEPES, 110 mm sucrose, and 0.1% (w/v) BSA; pH 7.1] using a pestle for Eppendorf tubes at a concentration of 1 mg of tissue (wet-weight) per 10 μl of MiR05. Respiration was measured in parallel to mitochondrial ROS production (O_2_^−^ and H_2_O_2_) at 37°C in the Oroboros O2k equipped with the O2K Fluo-LED2 Module (Oroboros Instruments). For mitochondrial ROS measurement, LEDs for green excitation were applied and a concentration of 1 mg of wet tissue per milliliter of MiR05 was added to final concentrations of 10 μm amplex red, 1 U/ml horseradish peroxidase, and 5 U/ml superoxide dismutase in 2 ml of MiR05 per O2K chamber. Calibration was performed by 5-μl titrations of 40 μm H_2_O_2_.

Respirational states were assessed using a standard high-resolution respirometry protocol: nicotinamide adenine dinucleotide (NADH)-pathway respiration in the LEAK (N*_L_*) state was initiated using malate (2 mm), pyruvate (10 mm), and glutamate (20 mm). Oxidative phosphorylation (N*_P_*) was stimulated by ADP (5 mm). Succinate (10 mm) addition yielded NADH- and succinate-linked respiration in oxidative phosphorylation (NS*_P_*) and in the uncoupled state (NS*_E_*) after titration (Δ0.5 μm) of carbonyl cyanide m-chlorophenyl hydrazine (CCCP). Complex I inhibition by rotenone (0.5 μm) allowed analysis of succinate-linked respiration in the uncoupled state (*SE*). All oxygen fluxes were corrected for residual (nonoxidative phosphorylation associated) oxygen consumption, ROX, after antimycin A was added. Respiratory acceptor control ratios (RCRs) were calculated as N*_L_*/NS*_P_*. Flux control ratios (FCRs) were assessed for NADH- (N*_P_*/NS*_E_*) succinate-driven respiration (*SE*/NS*_E_*). Mitochondrial ROS values are presented as mitochondrially generated O_2_^−^ and H_2_O_2_ per mg of wet weight and second, ROS values are corrected for background.

### Western blotting and fractionated sample preparations

After using the required volume for respirometry, phosphatase inhibitor mixes (Sigma, P5726 and P0044) and protease inhibitor mix (Sigma, P8340) were added to tissue homogenates at a ratio of 1:100, and then1 mM phenylmethylsulfonyl fluoride (PMSF) was added. Then, the samples were snap-frozen in liquid nitrogen and stored at −80°C. For extraction of Triton X-100 soluble and insoluble fractions, homogenates were diluted 1:1 in 1% Triton X-100/Tris-buffered saline (TBS; 50 mm Tris and 150 mm NaCl, pH 7.5) that included protease and phosphatase inhibitor mixes and PMSF at the same concentration as indicated above (total fractions). Homogenates were sonicated 10 times at a 0.5-s pulse (20% amplitude, Sonic Vibra Cell, Blanc Labo), and then they were incubated on ice for 30 min and centrifuged at 100,000 × *g* (30 min, 4°C). The supernatant was used as a soluble fraction, and the pellet was washed in 1% Triton X-100/TBS, sonicated as above, and centrifuged again at 100,000 × *g* (30 min, 4°C). The pellet (insoluble fraction) was resuspended in 2% sodium dodecyl sulfate (SDS)/TBS including protease and phosphatase inhibitor mixes and PMSF at the same concentration, as indicated above, and sonicated 15× at a 0.5-s pulse (20% amplitude). While sufficient tissue for tissue extractions was available for striatal samples (*N* = 8–9 per condition), two to three amygdala samples had to be pooled (at least *N* = 3 per condition).

Protein concentrations of different fractions were assessed using a BCA assay. A total of 15–20 μg of proteins was loaded on a 16% tricine gels and transferred onto a nitrocellulose membrane (Fisher Scientific) using a semidry system (Bio-Rad); 30 min of blocking in Odyssey blocking buffer (Li-Cor Biosciences) was followed by incubation overnight at 4°C with primary antibodies. Membranes were then washed in 0.01% (v/v) Tween 20 (Sigma-Aldrich) in PBS (PBS-T), and secondary antibodies were applied for 1 h at RT. After final washing in PBS-T, membranes were scanned using a Li-COR scanner (Li-Cor Biosciences).

### Antibodies

The antibodies used in this study are listed in Table 1.

**Table 1:** Antibodies

Type	Species	Specification	Concentration	Application
aSyn pS129	Mouse	Wako 014-20281	1:10,000 1:1000	IHC–DAB IHC
aSyn pS129 MJF-R13	Rabbit	Abcam 168381	1:750 1:4000	IHC WB
MAP2	Chicken	Abcam ab5392	1:2000	IHC
Ubiquitin 1	Mouse	Millipore MAB1510	1:500	IHC
p62	Mouse	Abcam ab56416	1:1000	IHC
TOM20	Mouse	Santa Cruz sc-17764	1:200 1:150	ICC IHC
aSyn 1-20	Rabbit	Eurogentec	1:1000	IHC
Anti-rabbit 647	Donkey	Invitrogen	1:800	IHC
Anti-mouse 488	Goat	Invitrogen	1:800	IHC
Anti-mouse 568	Goat	Invitrogen	1:800	IHC
Anti-chicken 488	Donkey	The Jackson Laboratory	1:500	IHC
ImmPRESS anti-mouse	Horse	Vector MP-7402	1 drop/section	IHC–DAB
SYN1 total α-synuclein	Mouse	BD 610787	1:1000	WB
OXPHOS	Mouse	Abcam ab110413	1:1000	WB
GAPDH/14C10	Rabbit	Cell Signaling, 2118S	1:2000	WB
Actin β	Mouse	Abcam ab6276	1:5000	WB
VDAC1	Mouse	Abcam ab14734	1:2000	WB
Anti-mouse Alexa Fluor Plus 800	Goat	Thermo Fisher Scientific A32730	1:20,000	WB
Anti-rabbit Alexa Fluor Plus 680	Goat	Thermo Fisher Scientific, A32734	1:20,000	WB

IHC, immunohistochemistry; ICC, immunocytochemistry; WB, Western blotting

### Statistics

Corticosterone versus vehicle and PFF injections versus PBS injections were compared in a 2 × 2 statistical design. Two-way ANOVAs were calculated, in cases for which Gaussian normality could be assumed. Normality was tested using the Anderson–Darling, D’Agostino–Pearson omnibus, Shapiro–Wilk, and Kolmogorov–Smirnov tests.

All absolute values and Western blotting values are presented as the mean ± SD. Normalized respirational and mitochondrial ROS values are presented as the mean ± SEM.

## Results

### Mitochondrial coupling efficiency is maintained in the presence of both seeded α-synuclein pathology and corticosterone induced alterations in body composition

**Figure 1.**
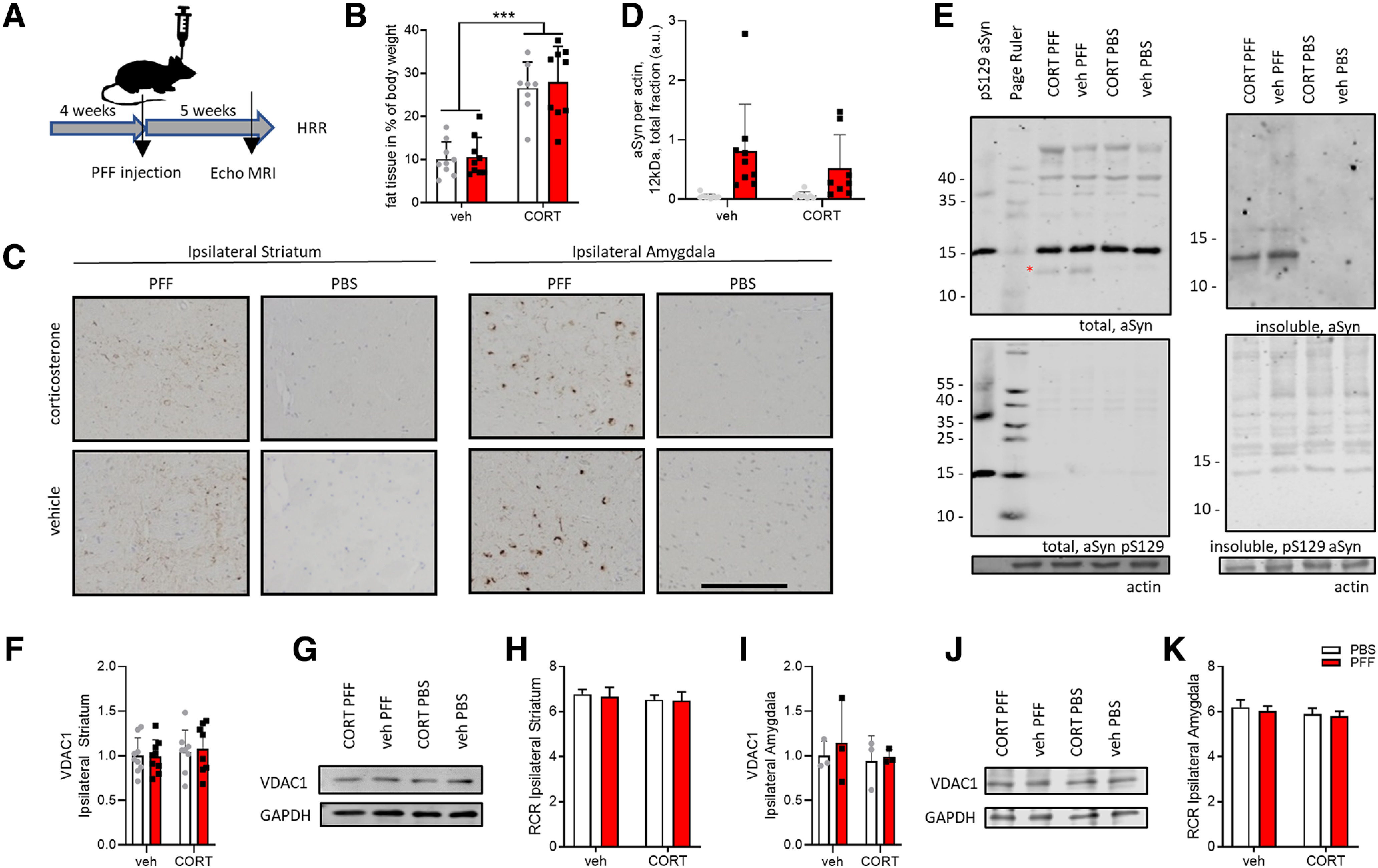
Effects of chronic corticosterone treatment and of α-synuclein (aSyn) inoculation. Animals were treated with corticosterone (CORT) or a solvent vehicle (veh) in the drinking water for one month, after which they were injected unilaterally in the dorsal striatum with 5 μg of PFFs or solvent (PBS). CORT/veh treatment was continued until approximately five weeks after surgery when the animals were killed (***A***). Fat per body weight was increased after eight weeks of CORT treatment (***B***). Six weeks after PFF injection α-synuclein pathology (α-synuclein pS129 immunostaining) was observed both in the striatum (mainly neuritic) and in the amygdala (mainly somatic; ***C***). The striatum and amygdala were dissected for high-resolution respirometry, and residual tissues were used for biochemical analyses. The PFF-injected striatum contained truncated α-synuclein (running at 12 kDa) in total homogenate, as quantified in ***D*** and represented in ***E*** (the asterisk indicates truncated 12 kDa α-synuclein), which was even more abundant in insoluble fractions (***E***). VDAC1 protein levels and mitochondrial RCRs were similar across groups both in the striatum (***F****–****H***) and in the amygdala (***I****–****K***); ***p < 0.001; two-way ANOVAs were performed. pS129 α-synuclein label for Western blotting lanes denotes recombinant protein controls. Scale bar: 200 μm (***C***).

First, we verified the effects of chronic corticosterone and injection of α-synuclein PFFs (Fig. 1*A*). Similar to our recent report (Burtscher et al., 2019), chronic corticosterone increased body fat tissue content, as assessed by Echo MRI (*F*_corticosterone(1,31)_ = 69,45, *p* < 0,0001; Fig. 1*B*), independently of PFF injection. α-Synuclein pS129 pathology was confirmed by histology in the ipsilateral striatum and amygdala (Fig. 1*C*). We observed similar pathology patterns in the PFF groups, regardless of corticosterone treatment, as reported previously (Burtscher et al., 2019).

Previous studies have shown that α-synuclein is subjected to proteolytic processing during or after its aggregation, which leads to both C- and N-terminal truncation of the protein (Baba et al., 1998; Gai et al., 1999; Grassi et al., 2018; Mahul-Mellier et al., 2018). Therefore, Western blot analysis was performed to investigate α-synuclein processing both in the striatum and amygdala five to six weeks after striatal PFF-injection. Several truncated forms of α-synuclein (running at ∼12 kDa), a signature of pathologic α-synuclein (Mahul-Mellier et al., 2018), were observed in ipsilateral (hemisphere of injection; Fig. 1*D*) total striatal fractions of all PFF conditions, although there was high variation in levels of these truncated forms of α-synuclein. Virtually all of the α-synuclein detected in all insoluble ipsilateral striatal fractions of PFF but not PBS conditions was truncated (Fig. 1*E*). Full-length α-synuclein (15 kDa) levels were similar in total striatal fractions. In fractionated amygdala samples, no or very little truncated α-synuclein was detected even in samples of PFF-injected mice. No α-synuclein pS129 signal was detected in any fraction by Western blotting, possibly due to dilution of the tissue, where the pathology was localized.

Next, we assessed general mitochondrial parameters, mitochondrial protein levels, and coupling efficiency. VDAC1 levels, as well as mitochondrial coupling efficiencies, estimated by mitochondrial RCRs, were similar across groups both in the striatum (Fig. 1*F–H*) and amygdala (Fig, 1*I–K*).

**Figure 2.**
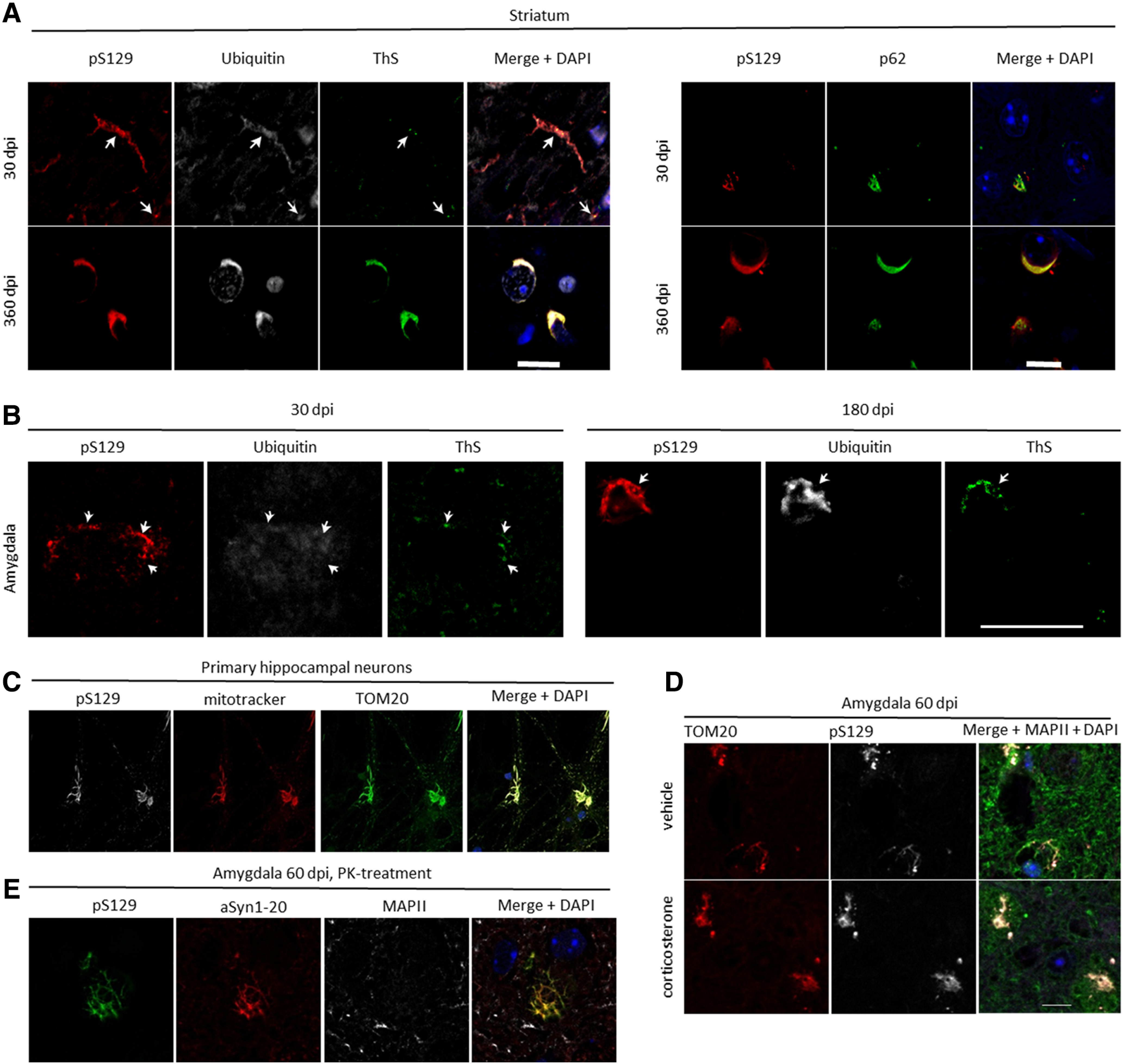
Characterization of α-synuclein pathology formation. In the striatum, pS129 immunoreactivity at 30 dpi revealed that the protein colocalized in peripheral cell compartments with ubiquitin, and there was some overlap with ThS and p62. This colocalization was more pronounced and perinuclear at 360 dpi (***A***). In the amygdala, similar colocalizations were observed at 30 dpi, and it was even more pronounced at 180 dpi (***B***). In primary hippocampal neurons 14 d after treatment with 70 nm PFFs, pS129 colocalized strongly with the mitochondrial markers TOM20 and Mito Tracker (***C***), with similar colocalizations being observed *ex vivo* at 60 dpi in the amygdala both in the chronic corticosterone and control condition (***D***). The aggregates in the amygdala were resistant to proteinase K (PK) at this time point (***E***). Green in the merged image in ***D*** corresponds to MAPII staining, and blue represents DAPI staining. Scale bars: 10 μm (***A***), 20 μm (***B***), and 10 μm (***D***, also for ***C***, ***E***). Arrows in ***A*** and ***B*** highlight examples of colocalizations.

Immunohistochemical analyses demonstrated an increasing immunoreactivity for the pS129-positive α-synuclein with other α-synuclein pathology markers (ubiquitin, ThS, and p62) over time in the striatum (Fig. 2*A*) and the amygdala (Fig. 2*B*).

To investigate potential colocalization of α-synuclein pathology with functional mitochondria, we labeled primary hippocampal neurons, in which α-synuclein pathology has been seeded 14 d before, with Mito Tracker. We indeed detected strong colocalization of pS129 with mitochondrial markers (Fig. 2*C*; Mahul-Mellier et al., 2020). Similar colocalization was also observed by co-immunostaining of brain sections for the outer mitochondrial membrane protein TOM20 and pS129 in neurons in the amygdala 60 dpi (Fig. 2*D*), which also contained proteinase K-resistant α-synuclein aggregations (Fig. 2*E*).

### Corticosterone treatment, but not α-synuclein pathology, alters mitochondrial respiration in the amygdala

**Figure 3.**
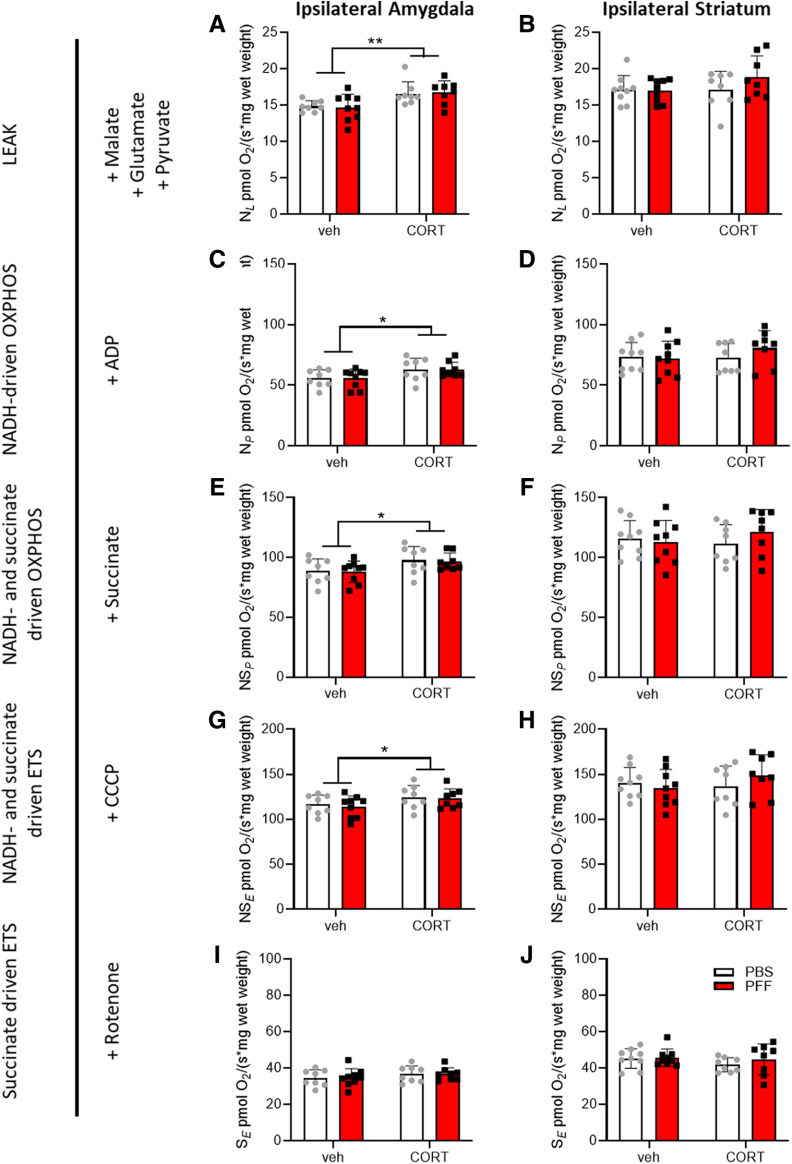
Testing mitochondrial functions *ex vivo*. The striatum and amygdala were dissected for high-resolution respirometry nine weeks after corticosterone (CORT) or vehicle (veh) treatment and/or after intrastriatal injection of 5 μg PFFs or solvent (PBS). Data from the hemispheres of injections (=ipsilateral) are depicted. The LEAK state in the presence of NADH substrates (N_L_) was measured (***A***, ***B***), followed by addition of ADP yielding NADH-driven oxidative phosphorylation (N_P_) in (***C***, ***D***) succinate to drive oxidative phosphorylation via both Complex I and Complex II (NS_P_; ***E***, ***F***) and CCCP to achieve the electron transport system maximum capacity (ETS, NS_E_; ***G***, ***H***). Subsequent inhibition of Complex I by rotenone resulted in succinate-driven, uncoupled respiration (*SE*; ***I***, ***J***). Respirational values of all states are given for the amygdala and striatum (hemisphere of injection = ipsilateral). The table to the right indicates the respirational state and substance additions according to the SUIT (substrate, uncoupler, inhibitor titration) protocol. Two-way ANOVA was performed; see Table 2 for detailed results. The main effects for CORT are indicated; *p < 0.05 and **p < 0.01.

Having validated that the applied treatments induced the expected phenotypes, high-resolution respirometry was applied concomitantly with amplex red fluorometry to assess mitochondrial respiration and ROS production in the striatum and amygdala tissues. In the presence of NADH-linked substrates and the absence of adenosine diphosphate (ADP, LEAK-state N*_L_*), respiration in the amygdala, was increased in corticosterone-treated mice (Fig. 3*A*), but not in the striatum (Fig. 3*B*). In the LEAK-state N*_L_* mitochondrial Complex I, shuttling of electrons from NADH generates a proton gradient that is not used for oxidative phosphorylation due to the unavailability of ADP. Thus, these conditions constitute a dissipative state of oxygen consumption that is primarily associated with heat production. Similarly, respiration was higher in the corticosterone condition in the NADH-driven oxidative phosphorylation state in the amygdala (saturating concentrations of ADP, N*_P_*; Fig. 3*C*) but not in the striatum (Fig. 3*D*). N*_P_* is a respirational state also linked to Complex I and driven by NADH-linked substrates, but the availability of ADP enables oxidative phosphorylation. Oxidative phosphorylation-mediated respiration was then increased by the addition of succinate, a substrate for mitochondrial Complex II, yielding NADH- and succinate-driven oxidative phosphorylation (NS*_P_*; Fig. 3*E*,*F*). The addition of a chemical uncoupling agent then allowed the induction of states in which respiration is uncoupled from oxidative phosphorylation. Similar to NS*_P_*, NADH- and succinate-driven uncoupled (NS*_E_*; Fig. 3*G*,*H*) respiration was also higher in the amygdala (Fig. 3*E*,*G*), but not in the striatum (Fig. 3*F*,*H*), in the corticosterone condition. Finally, in the succinate-driven, uncoupled state after Complex I inhibition (*SE*) using rotenone, no differences were observed in the amygdala (Fig. 3*I*) or in the striatum (Fig. 3*J*). Values for absolute respiration (per wet weight) and mitochondrial ROS production per brain region and normalizations to VDAC1 protein levels or total protein levels, as well as information on the statistical analyses are shown in Table 2.

**Table 2:** Summary of high-resolution respirometry data

		Striatum				
	State	LEAK	Oxidativephosphorylation (OXPHOS)		Uncoupled	
	Titration	Mal, Glu, Pyr	ADP	Succinate	CCCP	Rotenone
	Label	LEAK (N*_L_*)	N*_P_*	NS*_P_*	NS*_E_*	S*_E_*
Respiration perwet weight [pmol O_2_/(s*mg)]	Veh PBS	17.1 (2.0)	73.4 (12.0)	116.0 (15.0)	140.7 (16.9)	45.3 (5.4)
	CORT PBS	17.1 (2.5)	72.5 (12)	111.5 (16.1)	137.0 (22.2)	41.8 (3.9)
	Veh PFF	17.0 (1.6)	72.4 (14.0)	112.9 (18.1)	134.8 (20.6)	45.7 (4.7)
	CORT PFF	18.9 (2.9)	80,8 (14.4)	121.4 (19.0)	148.9 (22.7)	44.8 (8.5)
	Interaction	*F*_(1,30)_ = 1.395	*F*_(1,30)_ = 1.055	*F*_(1,30)_ = 1.225	*F*_(1,30)_ = 1.589	*F*_(1,30)_ = 0.384
		n.s.	n.s.	n.s.	n.s.	n.s.
	CORT factor	*F*_(1,30)_ = 1.410	*F*_(1,30)_ = 0.711	*F*_(1,30)_ = 0.116	*F*_(1,30)_ = 0.544	*F*_(1,30)_ = 1.176
		n.s.	n.s.	n.s.	n.s.	n.s.
	PFF factor	*F*_(1,30)_ = 1.049	*F*_(1,30)_ = 0.655	*F*_(1,30)_ = 0.341	*F*_(1,30)_ = 0.181	*F*_(1,30)_ = 0.725
		n.s.	n.s.	n.s.	n.s.	n.s.
FCRs (normalizationto NS*_E_*)	Veh PBS	0.12 (0.01)	0.52 (0.03)	0.82 (0.02)	1	0.32 (0.02)
	CORT PBS	0.13 (0.01)	0.53 (0.03)	0.82 (0.03)	1	0.31 (0.03)
	Veh PFF	0.13 (0.02)	0.53 (0.03)	0.84 (0.03)	1	0.34 (0.03)
	CORT PFF	0.13 (0.02)	0.54 (0.03)	0.82 (0.04)	1	0.30 (0.04)
	Interaction	*F*_(1,30)_ = 0.142	*F*_(1,30)_ = 0.044	*F*_(1,30)_ = 0.562	-	*F*_(1,30)_ = 1.902
		n.s.	n.s.	n.s.	-	n.s.
	CORT factor	*F*_(1,30)_ = 0.050	*F*_(1,30)_ = 0.622	*F*_(1,30)_ = 2.244	-	*F*_(1,30)_ = 6.469
		n.s.	n.s.	n.s.	-	*
	PFF factor	*F*_(1,30)_ = 0.622	*F*_(1,30)_ = 1.400	*F*_(1,30)_ = 0.395	-	*F*_(1,30)_ = 0.339
		n.s.	n.s.	n.s.	-	n.s.
Mitochondrial ROS [pmol/(s*mgwet weight)]	Veh PBS	0.11 (0.04)	0.06 (0.02)	-	-	0.39 (0.22)
	CORT PBS	0.12 (0.03)	0.06 (0.02)	-	-	0.37 (0.14)
	Veh PFF	0.10 (0.04)	0.07 (0.03)	-	-	0.36 (0.13)
	CORT PFF	0.14 (0.03)	0.09 (0.02)	-	-	0.35 (0.15)
	Interaction	*F*_(1,29)_ = 0.733	*F*_(1,28)_ = 0.784	-	-	*F*_(1,29)_ = 0.010
		n.s.	n.s.	-	-	n.s.
	CORT factor	*F*_(1,29)_ = 3.792	*F*_(1,28)_ = 0.848	-	-	*F*_(1,29)_ = 0.089
		n.s.	n.s.	-	-	n.s.
	PFF factor	*F*_(1,29)_ = 0.490	*F*_(1,28)_ = 2.699	-	-	*F*_(1,29)_ = 0.275
		n.s.	n.s.	-	-	n.s.
		Amygdala				
	State	LEAK	Oxidativephosphorylation		Uncoupled	
	Titration	Mal, Glu, Pyr	ADP	Succinate	CCCP	Rotenone
	Label	LEAK (N*_L_*)	N*_P_*	NS*_P_*	NS*_E_*	S*_E_*
Respiration per wetweight [pmol O_2_/(s*mg)]	Veh PBS	14.9 (0.7)	55.7 (7.1)	88.5 (15.9)	116.6 (10.5)	34.6 (4.5)
	CORT PBS	16.6 (1.6)	63.0 (9.3)	97.7 (11.5)	124.8 (12.8)	36.8 (4.5)
	Veh PFF	14.7 (1.8)	55.7 (7.6)	88.0 (9.1)	114.0 (12.2)	34.8 (4.8)
	CORT PFF	16.8 (1.6)	63.0 (6.3)	96.6 (7.2)	123.2 (10.8)	36.7 (3.5)
	Interaction	*F*_(1,29)_ = 0.088	*F*_(1,29)_ = 0	*F*_(1,29)_ = 0	*F*_(1,29)_ = 0.016	*F*_(1,29)_ = 0.012
		n.s.	n.s.	n.s.	n.s.	n.s.
	CORT factor	*F*_(1,29)_ = 12.670	*F*_(1,29)_ = 7.488	*F*_(1,29)_ = 6.983	*F*_(1,29)_ = 4.572	*F*_(1,29)_ = 1.786
		**	*	*	*	n.s.
	PFF factor	*F*_(1,29)_ = 0	*F*_(1,29)_ = 0	*F*_(1,29)_ = 0.060	*F*_(1,29)_ = 0.264	*F*_(1,29)_ = 0.004
		n.s.	n.s.	n.s.	n.s.	n.s.
FCRs (normalizationto NS*_E_*)	Veh PBS	0.13 (0.01)	0.48 (0.02)	0.76 (0.03)	1	0.30 (0.01)
	CORT PBS	0.13 (0.01)	0.50 (0.03)	0.78 (0.02)	1	0.29 (0.02)
	Veh PFF	0.13 (0.01)	0.48 (0.03)	0.77 (0.02)	1	0.30 (0.01)
	CORT PFF	0.14 (0.02)	0.51 (0.03)	0.78 (0.03)	1	0.30 (0.02)
	Interaction	*F*_(1,29)_ = 0.040	*F*_(1,29)_ = 0.021	*F*_(1,29)_ = 0.351	-	*F*_(1,29)_ = 0.166
		n.s.	n.s.	n.s.	-	n.s.
	CORT factor	*F*_(1,29)_ = 1.711	*F*_(1,29)_ = 6.773	*F*_(1,29)_ = 3.784	-	*F*_(1,29)_ = 0.250
		n.s.	*	n.s.	-	n.s.
	PFF factor	*F*_(1,29)_ = 0.163	*F*_(1,29)_ = 0.973	*F*_(1,29)_ = 0.840	-	*F*_(1,29)_ = 0.891
		n.s.	n.s.	n.s.	-	n.s.
Mitochondrial ROS [pmol/(s*mgwet weight)]	Veh PBS	0.13 (0.03)	0.09 (0.03)	-	-	0.41 (0.17)
	CORT PBS	0.13 (0.03)	0.09 (0.04)	-	-	0.44 (0.18)
	Veh PFF	0.12 (0.03)	0.09 (0.02)	-	-	0.39 (0.13)
		Amygdala				
	State	LEAK	Oxidativephosphorylation		Uncoupled	
	Titration	Mal, Glu, Pyr	ADP	Succinate	CCCP	Rotenone
	Label	LEAK (N*_L_*)	N*_P_*	NS*_P_*	NS*_E_*	S*_E_*
	CORT PFF	0.15 (0.03)	0.09 (0.02)	-	-	0.40 (0.19)
	Interaction	*F*_(1,28)_ = 1.142	*F*_(1,27)_ = 0.095	-	-	*F*_(1,28)_ = 0.026
		n.s.	n.s.	-	-	n.s.
	CORT factor	*F*_(1,28)_ = 1.423	*F*_(1,27)_ = 0.203	-	-	*F*_(1,28)_ = 0.089
		n.s.	n.s.	-	-	n.s.
	PFF factor	*F*_(1,28)_ = 0.009	*F*_(1,27)_ = 0.159	-	-	*F*_(1,28)_ = 0.210
	n.s.	n.s.	-	-	n.s.

Mean values (SD) are given for the different conditions with details on statistical tests (two-way ANOVAs) in the rows below them; *p < 0.05 and **p < 0.01.

**Figure 4.**
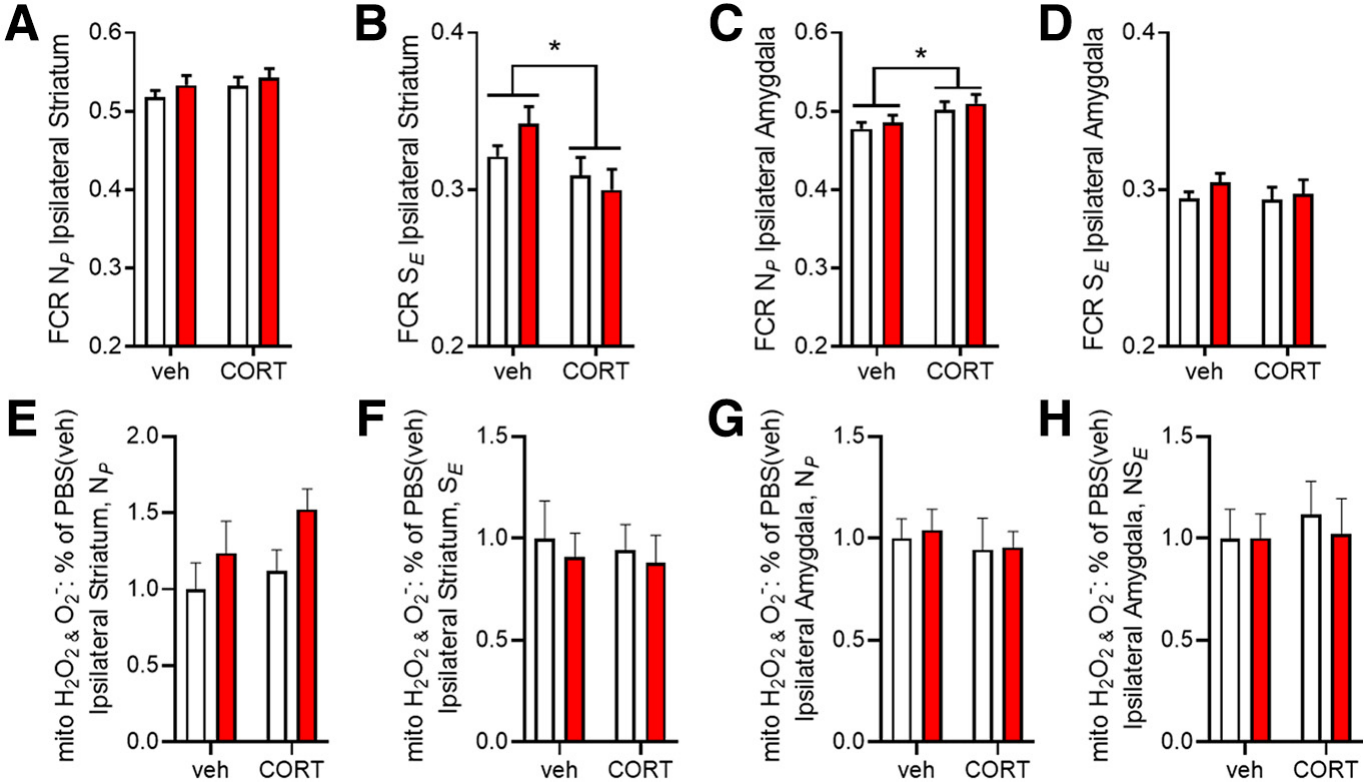
FCRs and mitochondrial ROS. FCRs are presented for the striatum (***A***, ***B***) and amygdala (***C***, ***D***). Mitochondrial ROS production was similar across groups in both in the striatum (***E***, ***F***) and the amygdala (***G***, ***H***). Two-way ANOVA was performed; see Table 2 for detailed results. The main effects of CORT are indicated; *p < 0.05.

To assess qualitative changes in respiration control, FCRs were calculated. While there were no differences in NADH-driven (N*_P_*) FCRs in the presence of ADP between corticosterone and vehicle control groups (Fig. 4*A*), FCR was reduced for the uncoupled, succinate-driven state (N*_E_*) in corticosterone conditions (*F*_corticosterone(1,30)_ = 6634, *p* = 0,015) in the ipsilateral striatum (Fig. 4*B*). In the amygdala, the NADH-driven (N*_P_*) FCR was higher in corticosterone conditions (*F*_corticosterone(1,29)_ = 5907, *p* = 0,021; Fig. 4*C*), but there were no significant differences compared with vehicle control groups in noncoupled, succinate-driven (N*_E_*) FCR (Fig. 4*D*).

We hypothesized that hyperactivity of the amygdala following chronic corticosterone treatment, or exposure to α-synuclein pathology might result in increased mitochondrial ROS generation. However, amplex red fluometry revealed no differences in mitochondrial ROS production either in the striatum (Fig. 4*E*,*F*) or amygdala (Fig. 4*G*,*H*).

## Discussion

Following up on our findings that α-synuclein pathology in mouse amygdala did not cause strong behavioral deficits (Burtscher et al., 2019), we investigated here, whether α-synuclein pathology, as defined above, was sufficient to cause mitochondrial dysfunction in the amygdala or at the site of injection (striatum). In the applied model, histologic staining of pS129 α-synuclein showed a peak in levels between one and three months in the amygdala that decreased thereafter. Conversely, in the striatum, the pathology does not decrease but changes from predominantly neuritic localization at the investigated time point to successively more perinuclear localization at a later time points (Burtscher et al., 2019). In the present study, we were interested in the interplay between mitochondrial dysfunction and α-synuclein pathology at the time point where pS129 α-synuclein immunoreactivity peaks in the amygdala. Therefore, we cannot rule out progressive mitochondrial dysfunction at later time points in this model, e.g., in the striatum.

Impaired electron transport chain function due to the presence or accumulation of aggregated forms of α-synuclein has been repeatedly reported in cellular models (Chinta et al., 2010; Reeve et al., 2015; Tapias et al., 2017; Kim et al., 2018; Wang et al., 2019), especially at late stages of aggregation formation (Mahul-Mellier et al., 2020). However, functional assessment of mitochondrial respiration in *in vivo* models of α-synuclein seeding and pathology spreading remains lacking. Therefore, we aimed to study mitochondrial respiration directly on fresh brain tissue from regions affected by α-synuclein pathology using *ex vivo* respirometry, which is the gold standard used to assess mitochondrial function.

We did not observe differences in absolute respiration related to α-synuclein pathology both in the amygdala and the striatum. In contrast, chronic corticosterone treatment (associated with apparent depression-like phenotypes but not with significantly changed α-synuclein pathology; Burtscher et al., 2019) increased mitochondrial respiration in the amygdala. The observed elevated respiration is in line with previous reports that systemic administration of corticosterone can increase the activity of the basolateral amygdala (Kavushansky and Richter-Levin, 2006). Furthermore, the respirometry protocols applied here allowed the identification of primarily NADH-linked substrates that drive this increased respiration.

To investigate potential qualitative changes in respiration patterns, we calculated FCRs (Gnaiger, 2009) and detected a reduced succinate-driven FCR in corticosterone conditions in the striatum. A higher succinate-driven FCR after the surgical intervention in the absence of exogenous corticosterone might be an adaptative response of the damaged tissue (Lukyanova and Kirova, 2015; Weilnau et al., 2018). Protein aggregation is believed to induce conditioning-like cellular adaptations (Mao and Crowder, 2010). Liu and colleagues reported less efficient preconditioning-related adaptations if animals were treated with exogenous corticosterone (Liu and Zhou, 2012) and the HPA (hypothalamus-pituitary gland-adrenal gland) axis is intimately linked with molecular adaptation to stressors (Rybnikova and Samoilov, 2015), which might explain the absence of succinate-driven FCR upregulation in the corticosterone condition. The NADH-driven FCR in the oxidative phosphorylation state was higher in the corticosterone conditions in the amygdala, confirming the focal role of Complex I in the observed effect of enhanced respiration after chronic corticosterone administration.

**Figure 5.**
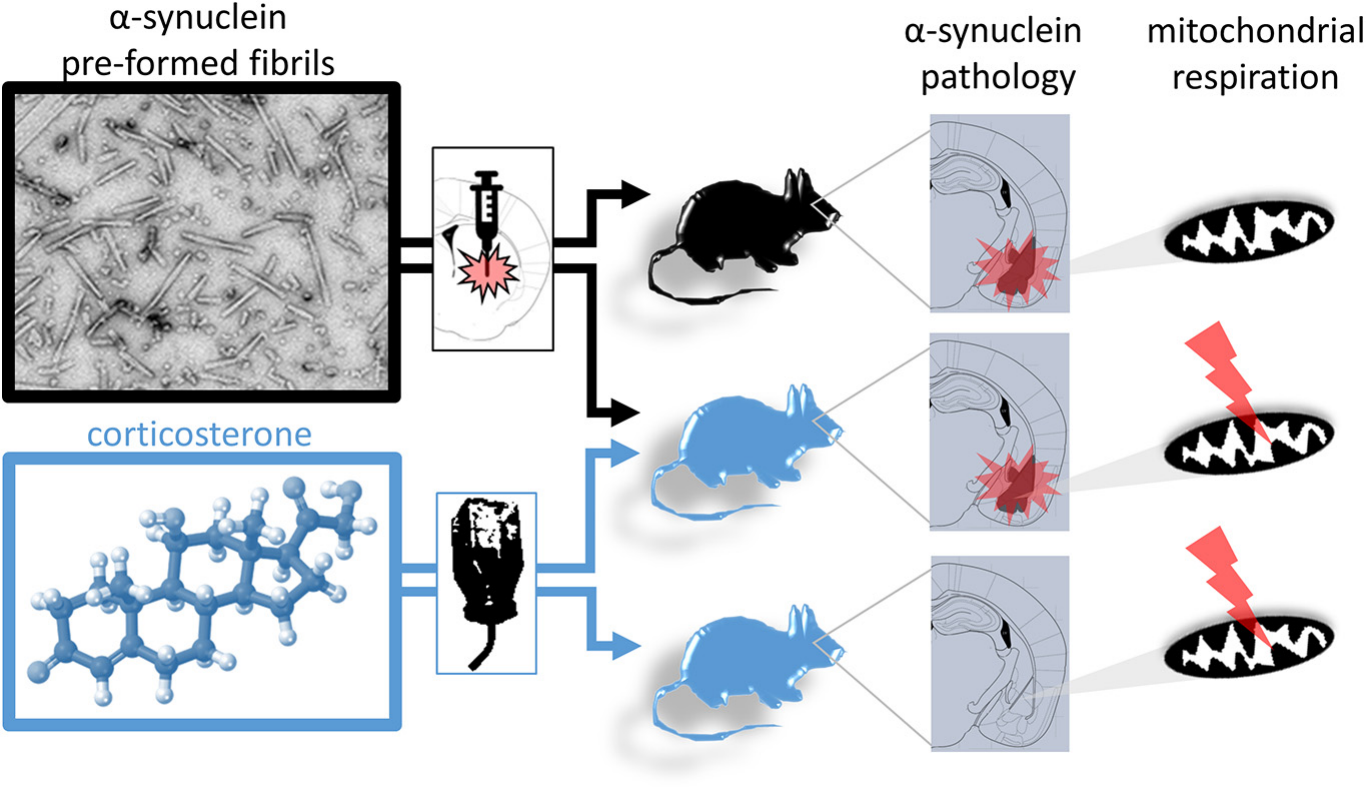
Working model. While chronic corticosterone induced respirational hyperactivity in the amygdala and PFF inoculation resulted in pronounced levels of pathologic α-synuclein, no deficits of mitochondrial respiration were observed as a result of α-synuclein pathology formation.

Misfolded α-synuclein has previously been associated with oxidative stress (Hsu et al., 2000). Additionally, chronic corticosterone treatment has been demonstrated to result in a dysregulated oxidative balance (Liu and Zhou, 2012). Therefore, we tested whether α-synuclein pathology or chronic corticosterone resulted in increased mitochondrial ROS production in our model, which was not the case. In summary, these results suggest that α-synuclein pathology as defined here (immunoreactivity to pS129 and C-terminal truncated, insoluble forms of α-synuclein) and five to six weeks postinjection is not sufficient to induce clear mitochondrial dysfunction in the mouse amygdala and striatum. The additional challenge of the amygdala circuits with corticosterone did not result in mitochondrial dysfunction, although it might have blocked mitochondrial adaptations in response to α-synuclein pathology formation. A summary of the reported findings is given in Figure 5.

Importantly, as we did not observe pS129 α-synuclein in the striatum or amygdala by Western blotting five to six weeks after PFF injection, even in the insoluble fractions, we assume that at this time point pS129-positive, insoluble aggregates were too rare to be detected. Conversely, by immunostaining pS129 α-synuclein was readily detected. This could mean that pS129-positive α-synuclein pathology precedes the formation of insoluble aggregates, which is consistent with our recent study on the successive maturation of pS129 α-synuclein-positive aggregates into Lewy body-like structures (Mahul-Mellier et al., 2020).

A limitation of the study performed here is the possibility that mitochondrial dysfunction was not detected because the number of affected neurons was too low for changes to be recognized. While we cannot exclude this possibility, which warrants further investigation, we demonstrated that overall brain region respirational patterns were also maintained in the presence of dense pS129 α-synuclein pathology. On the other hand, the chronic supply of exogenous corticosterone increased mitochondrial respiration in the amygdala independent of pS129 α-synuclein pathology.

The primary aim of this study was to investigate whether α-synuclein pathology immediately results in mitochondrial dysfunction. We deem that for this purpose, the inclusion of only male mice was sufficient, given that the incidence of synucleinopathies is higher in men than it is in women (Savica et al., 2013). However, this divergent incidence warrants specific investigation of sex-specific differences in the interaction of α-synuclein pathology and mitochondrial dysfunction in future studies.

## Conclusions

Both α-synuclein pathology and mitochondrial dysfunction have been implicated in the pathogenesis of PD and other synucleinopathies. Despite recent advances in the elucidation of the molecular underpinnings of both processes, it remains unclear how α-synuclein pathology influences mitochondrial function *in vivo* and vice versa and whether one or both of these hallmarks are sufficient to cause neurodegeneration and the manifestation of clinical symptoms. Our results suggest that the prominent α-synuclein pathology observed at five to six weeks postinoculation with PFFs and, as defined above, is not sufficient to induce mitochondrial dysfunction. Although significant cell loss in the substantia nigra in this model is usually only detected six months after intrastriatal PFF injection (Luk et al., 2012), we expected pronounced levels of pathologic α-synuclein early after seeding to have effects on mitochondrial function. A recent report demonstrated a non-significant trend toward a reduction in neuron numbers in the basolateral amygdala only six months after bilateral injection of high PFF concentrations (Stoyka et al., 2020).

Our findings suggest that mitochondrial dysfunction may occur later, possibly during post α-synuclein aggregation events linked to the transition from pS129 immunoreactive filamentous aggregates to LBs, which also involve the recruitment of mitochondria and other membranous organelles into LBs (for review, see Lashuel, 2020). Indeed, recent studies in primary neuron models have shown that recruitment of mitochondria and mitochondrial dysfunction occur primarily at later stages of LB formation and maturation (Mahul-Mellier et al., 2020). Together, these findings highlight the critical importance of revisiting the interplay between α-synuclein and mitochondria at the various stages on the pathway to LB formation and their roles in LB formation and maturation and neurodegeneration in this model and other animal models of PD pathology and synucleinopathies.

Our findings demand caution regarding the translational validity of this PFF injection model, given the weak symptomatology, in particular of non-motor symptoms, even at the peak of α-synuclein pathology in this model (one to three months after injection of PFFs; Burtscher et al., 2019). It is noteworthy that it appears to be challenging to reproduce motor symptoms in this PFF inoculation model, even at the late stages of pathology formation. For example, recently Henderson et al. (2019) reported no deficits in rotarod-assessed motor coordination even up to nine months after PFF injection, despite this being one of the most replicated features in this model (Luk et al., 2012; Kim et al., 2019). We also warn about potential publication bias, with negative reports on this model not being publicly shared and discussed, and we hope that our report encourages other scientists to share and discuss their negative findings.

## Synthesis

Reviewing Editor: Dritan Agalliu, Columbia University Medical Center Decisions are customarily a result of the Reviewing Editor and the peer reviewers coming together and discussing their recommendations until a consensus is reached. When revisions are invited, a fact-based synthesis statement explaining their decision and outlining what is needed to prepare a revision will be listed below. The following reviewer(s) agreed to reveal their identity: Ashley Harms.

The study by Burtscher et al investigated the effect of early aSyn pathology on mitochondrial respiration, ROS generation and protein abundance, which have long been suggested to be the hallmark of PD pathogenesis. Using a combination of aSyn PFF and chronic corticosterone administration the authors demonstrate that at 5 weeks post-seeding there is no detectable mitochondrial pathology in the striatum and amygdala of mice.

The reviews are mixed for this manuscript, but despite the negative conclusions of the study, experiments are well designed and the data are thoroughly presented.

Reviewer # 1

Major concerns:

1. The manuscript is difficult to read and the justification for experimental design and measurement of endpoints are difficult to follow. The results section is short and lacks detail regarding what the mitochondrial respiration parameters indicate. Please revise the manuscript to expand on the experimental design and the rationale for the study.

2. Why were only male mice used in the study? What is the rationale for this choice?

3. Would the authors see similar results with higher concentrations of pre-formed fibrils?

Minor:

1. There are several issues with references within the manuscript including the lab’s previous paper. Please fix the issues with the references.

2. There are several typographical errors including one in the abstract “Although injection of α-synuclein preformed fibrils into the striatum resulted in pronounced α- synuclein pathology in both striatum and amygdala, mitochondrial respiration in these brain regions was altered in neither chronic corticosterone nor control conditions.” Please have an English speaker proofread the manuscript.

Reviewer # 2

Minor points:

1. Line 45. Instead of “was altered” should read “was not altered”.

2. Line 161. “...primary cultures were treated with 70 nM of PFFs.” How was the concentration of PFF calculated? Or it should be ng instead of nM?

3. Line 625. Here and in other Figure legends, please indicate the statistical test that was used.

4. Lines 636-939. Reference to panels D and E are swapped.
